# Supplementary material for: An intrinsic cell cycle timer terminates limb bud outgrowth
Source: eLife. 2018 Sep 3;7:e37429. doi: 10.7554/eLife.37429 (PMC6143340; doi:10.7554/eLife.37429)
Supplement: Figure 3—source data 1. [file elife-37429-fig3-data1.pdf]

Source data for flow cytometry in Figure 3k

HH20 left+8h

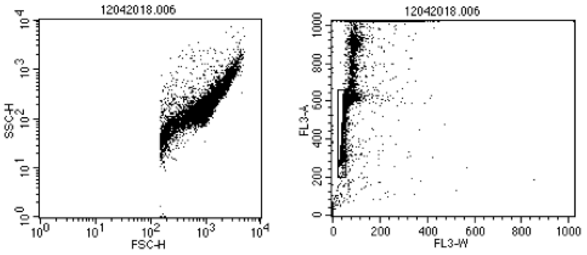

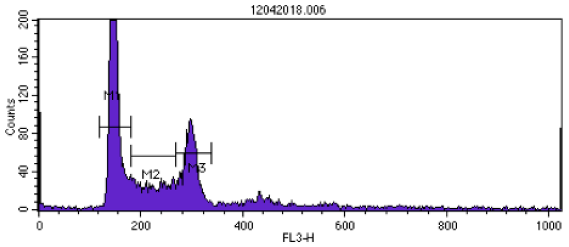

Histogram Statistics

File: 12042018.006
Sample ID:
Panel: Untitled Acquisition Tube List
Gate: No Gate
Total Events: 13976

Log Data Units: Linear Values
Tube: Untitled
Acquisition Date: 29-Jun-18
Gated Events: 13976
X Parameter: FL3-H (Linear)

Marker	Left, Right	Events	% Gated	% Total	Mean	Geo Mean	CV	Median	Peak Ch
All	0, 1023	13976	100.00	100.00	230.66	140.14	75.52	162.00	0
M1	119, 181	6324	45.25	45.25	148.90	148.63	6.25	147.00	146
M2	181, 269	1903	13.62	13.62	224.68	223.10	11.82	224.00	262
M3	269, 338	2683	19.20	19.20	296.90	296.61	4.50	297.00	297

HH20+PBS+8h

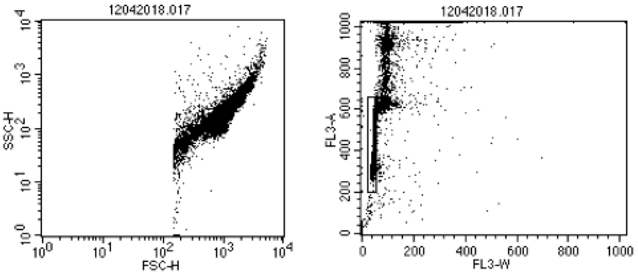

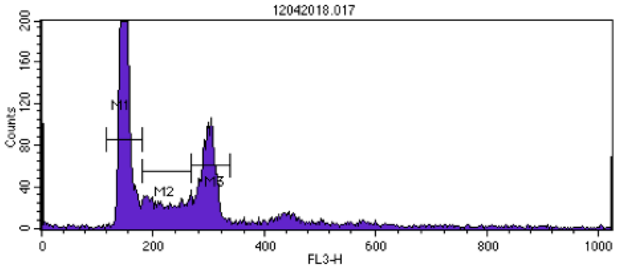

Histogram Statistics

File: 12042018.017
Sample ID:
Panel: Untitled Acquisition Tube List
Gate: No Gate
Total Events: 13745

Log Data Units: Linear Values
Tube: Untitled
Acquisition Date: 29-Jun-18
Gated Events: 13745
X Parameter: FL3-H (Linear)

Marker	Left, Right	Events	% Gated	% Total	Mean	Geo Mean	CV	Median	Peak Ch
All	0, 1023	13745	100.00	100.00	233.19	152.61	71.09	164.00	0
M1	116, 181	6327	46.03	46.03	149.82	149.58	5.76	148.00	147
M2	181, 269	1864	13.56	13.56	224.11	222.49	12.02	223.00	269
M3	269, 338	2826	20.56	20.56	298.66	298.36	4.51	299.00	305

HH20 left+8h

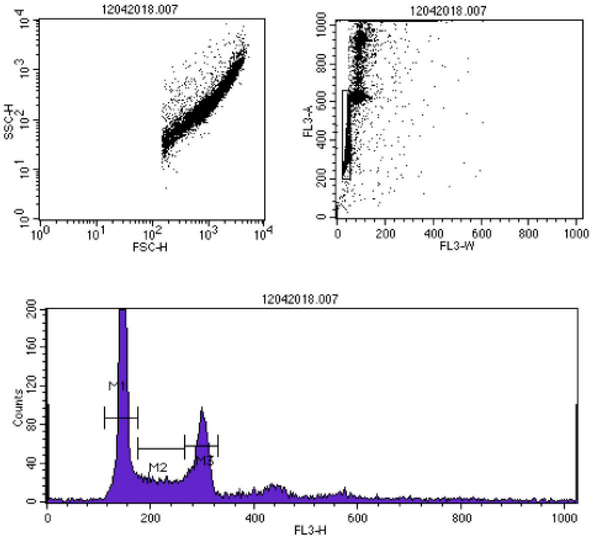

Histogram Statistics

File: 12042018.007
Sample ID:
Panel: Untitled Acquisition Tube List
Gate: No Gate
Total Events: 14020

Log Data Units: Linear Values
Tube: Untitled
Acquisition Date: 29-Jun-18
Gated Events: 14020
X Parameter: FL3-H (Linear)

Marker	Left, Right	Events	% Gated	% Total	Mean	Geo Mean	CV	Median	Peak Ch
All	0, 1023	14020	100.00	100.00	257.79	185.37	74.56	171.00	147
M1	111, 176	6531	46.58	46.58	147.41	147.16	5.87	147.00	147
M2	176, 266	1711	12.20	12.20	220.03	218.36	12.30	219.00	197
M3	266, 331	2703	19.28	19.28	296.79	296.47	4.59	298.00	299

HH20+Nog+8h

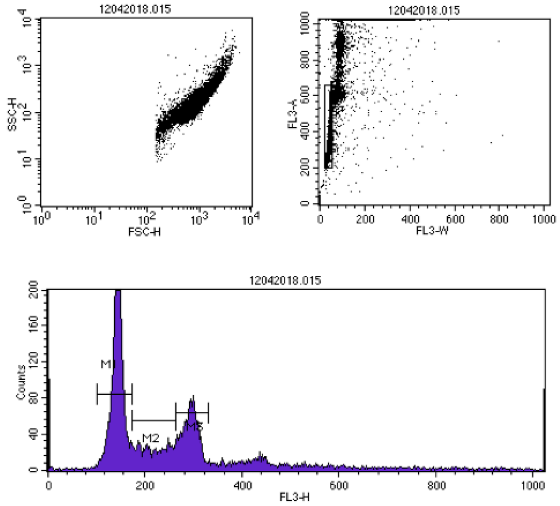

Histogram Statistics

File: 12042018.015
Sample ID:
Panel: Untitled Acquisition Tube List
Gate: No Gate
Total Events: 13352

Log Data Units: Linear Values
Tube: Untitled
Acquisition Date: 29-Jun-18
Gated Events: 13352
X Parameter: FL3-H (Linear)

Marker	Left, Right	Events	% Gated	% Total	Mean	Geo Mean	CV	Median	Peak Ch
All	0, 1023	13352	100.00	100.00	241.23	186.64	70.89	163.00	146
M1	101, 173	6558	49.12	49.12	143.37	142.92	7.74	144.00	146
M2	173, 264	1889	14.15	14.15	219.74	218.00	12.48	220.00	249
M3	264, 331	2643	19.79	19.79	293.33	292.94	5.12	294.00	299

HH20 left+8h

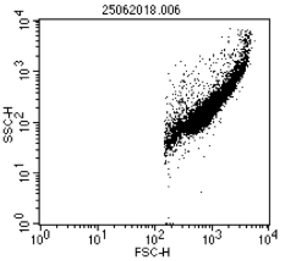

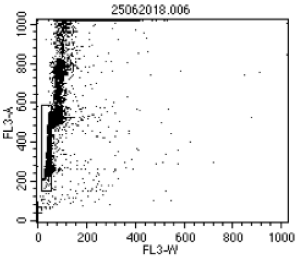

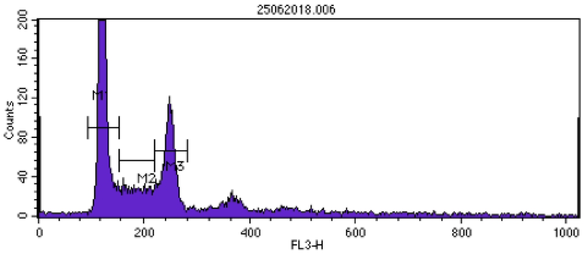

Histogram Statistics

File: 25062018.006
Sample ID:
Panel: Untitled Acquisition Tube List
Gate: No Gate
Total Events: 13961

Log Data Units: Linear Values
Tube: Untitled
Acquisition Date: 25-Jun-18
Gated Events: 13961
X Parameter: FL3-H (Linear)

Marker	Left, Right	Events	% Gated	% Total	Mean	Geo Mean	CV	Median	Peak Ch
All	0, 1023	13961	100.00	100.00	219.25	156.77	83.29	139.00	121
M1	93, 152	6744	48.31	48.31	122.77	122.52	6.51	122.00	121
M2	152, 220	1569	11.24	11.24	186.44	185.35	10.78	186.00	161
M3	220, 282	2773	19.86	19.86	246.45	246.16	4.86	247.00	247

HH24g+ PBS+8h

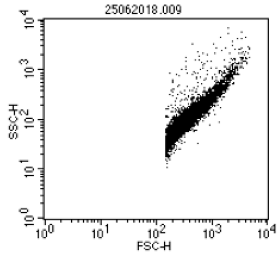

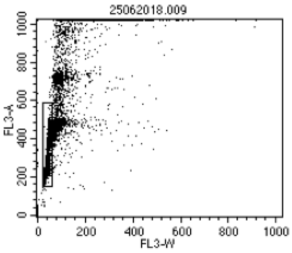

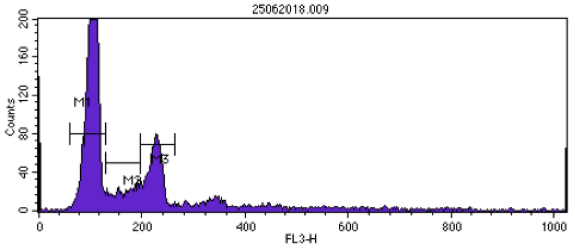

Histogram Statistics

File: 25062018.009
Sample ID:
Panel: Untitled Acquisition Tube List
Gate: No Gate
Total Events: 12892

Log Data Units: Linear Values
Tube: Untitled
Acquisition Date: 25-Jun-18
Gated Events: 12892
X Parameter: FL3-H (Linear)

Marker	Left, Right	Events	% Gated	% Total	Mean	Geo Mean	CV	Median	Peak Ch
All	0, 1023	12892	100.00	100.00	175.15	139.02	84.74	112.00	109
M1	59, 129	7868	61.03	61.03	104.01	103.48	9.67	106.00	109
M2	129, 196	1100	8.53	8.53	166.24	164.95	12.30	170.00	191
M3	196, 264	2289	17.76	17.76	224.42	224.03	5.90	225.00	227

HH24g+Nog+8h

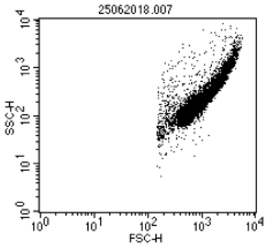

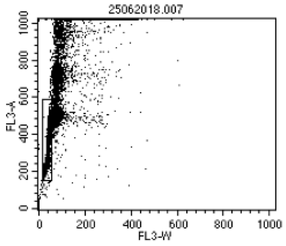

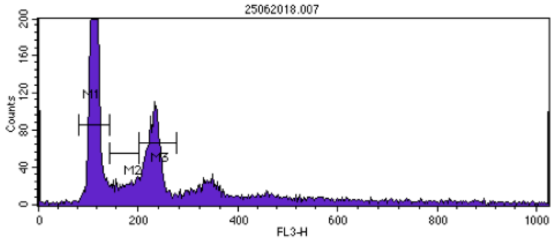

Histogram Statistics

File: 25062018.007
Sample ID:
Panel: Untitled Acquisition Tube List
Gate: No Gate
Total Events: 15467

Log Data Units: Linear Values
Tube: Untitled
Acquisition Date: 25-Jun-18
Gated Events: 15467
X Parameter: FL3-H (Linear)

Marker	Left, Right	Events	% Gated	% Total	Mean	Geo Mean	CV	Median	Peak Ch
All	0, 1023	15467	100.00	100.00	236.64	170.17	85.96	150.00	114
M1	80, 142	7156	46.27	46.27	113.10	112.80	7.29	113.00	114
M2	142, 202	1173	7.58	7.58	173.46	172.49	10.51	175.00	199
M3	201, 276	3124	20.20	20.20	231.09	230.61	6.47	231.00	233

HH20 left+8h

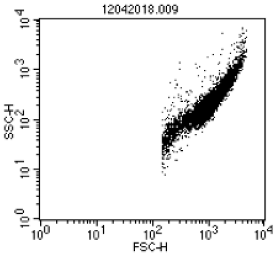

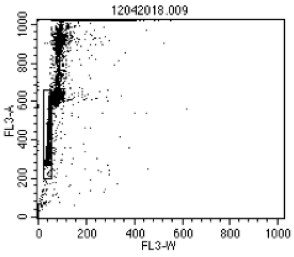

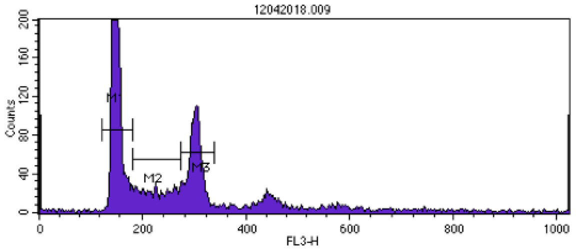

Histogram Statistics

File: 12042018.009
Sample ID:
Panel: Untitled Acquisition Tube List
Gate: No Gate
Total Events: 13624

Log Data Units: Linear Values
Tube: Untitled
Acquisition Date: 29-Jun-18
Gated Events: 13624
X Parameter: FL3-H (Linear)

Marker	Left, Right	Events	% Gated	% Total	Mean	Geo Mean	CV	Median	Peak Ch
All	0, 1023	13624	100.00	100.00	252.65	181.26	71.46	176.00	0
M1	121, 181	6279	46.09	46.09	149.97	149.74	5.73	148.00	148
M2	181, 274	1740	12.77	12.77	227.33	225.61	12.26	226.50	224
M3	274, 338	2851	20.93	20.93	301.10	300.85	4.07	301.00	305

HH20+Bmp2 low+8h

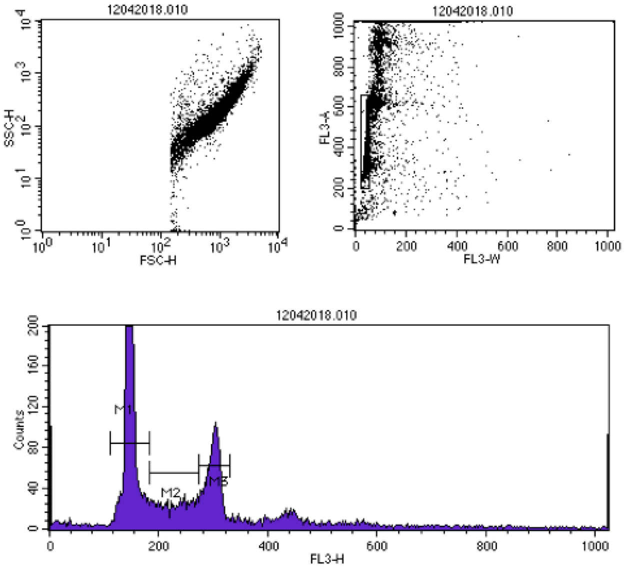

Histogram Statistics

File: 12042018.010
Sample ID:
Panel: Untitled Acquisition Tube List
Gate: No Gate
Total Events: 14616

Log Data Units: Linear Values
Tube: Untitled
Acquisition Date: 29-Jun-18
Gated Events: 14616
X Parameter: FL3-H (Linear)

Marker	Left, Right	Events	% Gated	% Total	Mean	Geo Mean	CV	Median	Peak Ch
All	0, 1023	14616	100.00	100.00	240.43	170.22	72.05	162.00	0
M1	111, 183	6984	47.78	47.78	148.40	148.06	6.86	147.00	147
M2	183, 274	1917	13.12	13.12	229.81	228.12	12.02	231.00	249
M3	274, 331	2649	18.12	18.12	299.44	299.21	3.96	300.00	304

HH20+Bmp2 high+8h

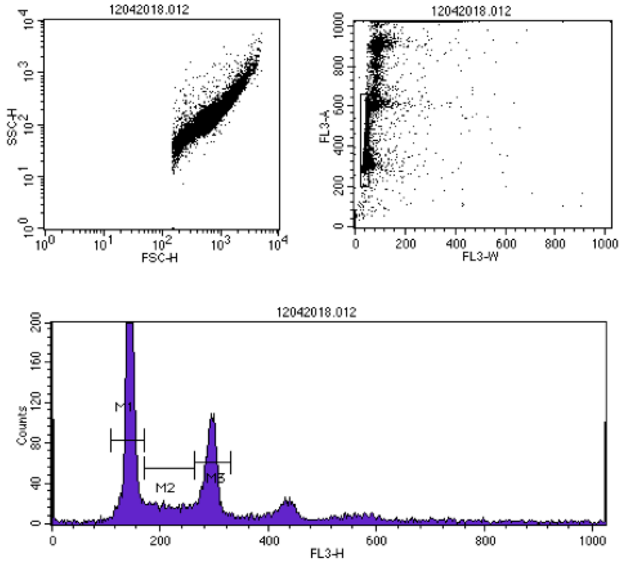

Histogram Statistics

File: 12042018.012
Sample ID:
Panel: Untitled Acquisition Tube List
Gate: No Gate
Total Events: 14841

Log Data Units: Linear Values
Tube: Untitled
Acquisition Date: 29-Jun-18
Gated Events: 14841
X Parameter: FL3-H (Linear)

Marker	Left, Right	Events	% Gated	% Total	Mean	Geo Mean	CV	Median	Peak Ch
All	0, 1023	14841	100.00	100.00	256.20	193.63	76.51	152.00	144
M1	109, 171	7623	51.36	51.36	144.11	143.91	5.22	144.00	144
M2	171, 264	1358	9.15	9.15	216.76	214.96	12.85	216.00	207
M3	264, 331	2720	18.33	18.33	293.28	293.02	4.21	294.00	295
