# Supplementary figures and images for: An intrinsic cell cycle timer terminates limb bud outgrowth
Source: eLife. 2018 Sep 3;7:e37429. doi: 10.7554/eLife.37429 (PMC6143340; doi:10.7554/eLife.37429)

Source data for flow cytometry in Figure 4k

HH27 left+6h

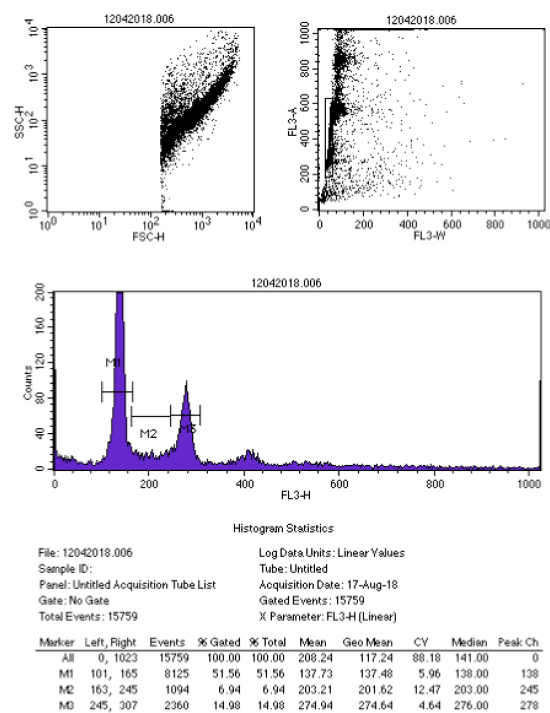

HH27+PBS+6h

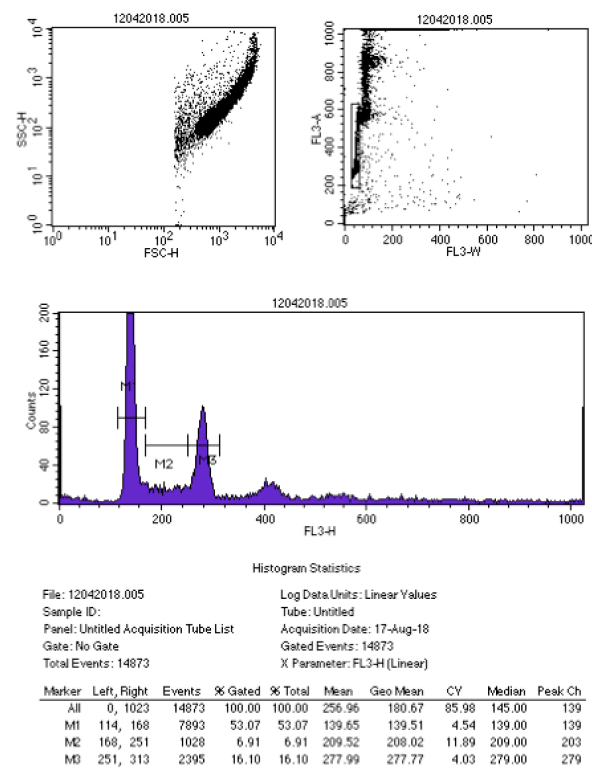

HH27 left+6h

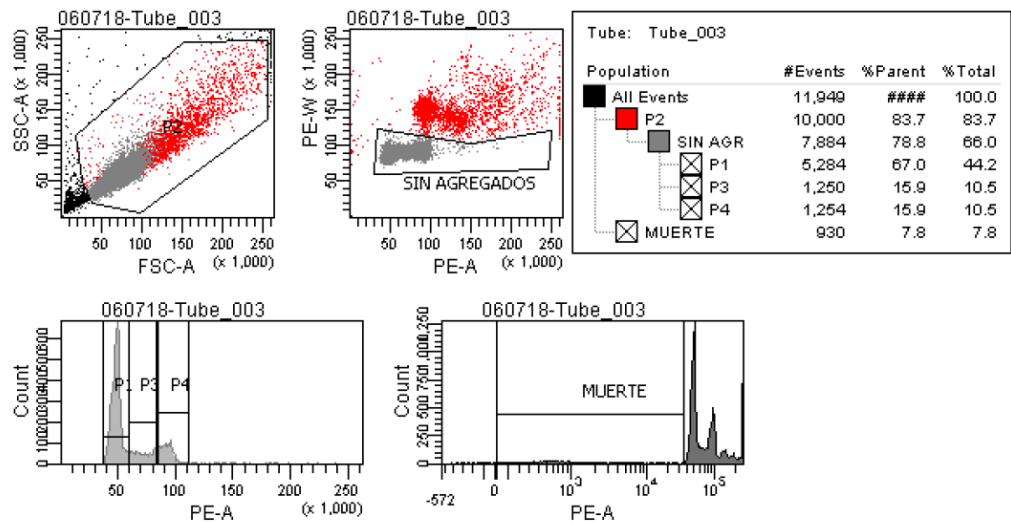

HH27+FGF8+6h

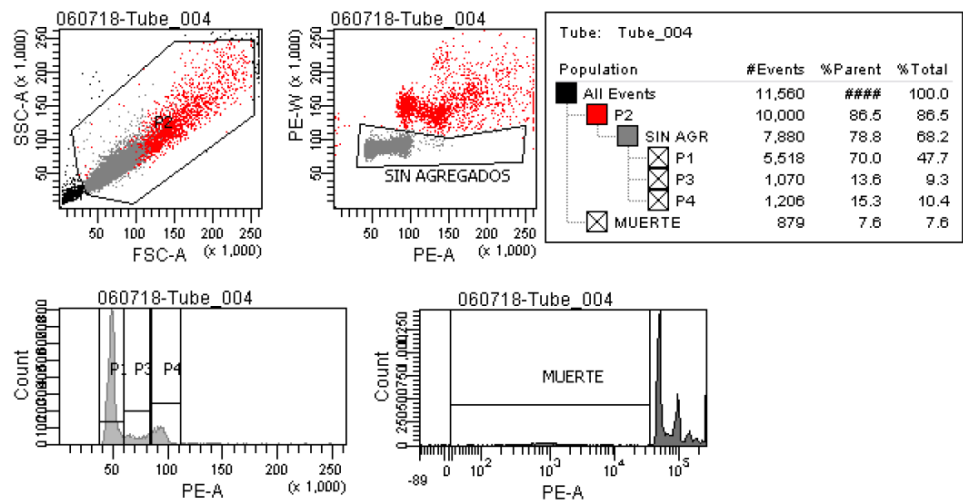

Supplement: Figure 4—source data 1. [file elife-37429-fig4-data1.pdf]
